# Application of Metabolomics in Defence Responses of *Brassica* Crops

**DOI:** 10.3390/metabo16080563

**Published:** 2026-08-10

**Authors:** Yufei Li, Junxing Lu

**Affiliations:** Chongqing Key Laboratory of Plant Environmental Adaptations Biology, College of Life Sciences, Chongqing Normal University, Chongqing 401331, China; 2024210513014@stu.cqnu.edu.cn

**Keywords:** *Brassica* crops, metabolomics, secondary metabolites, plant defence, stress responses

## Abstract

*Brassica* crops, encompassing globally important vegetables and oilseeds, face severe threats from diverse biotic and abiotic stresses. Plant secondary metabolites constitute the chemical foundation of defence, and metabolomics has emerged as an effective systems biology tool for comprehensively dissecting stress-induced metabolic changes. Recent progress in applying metabolomics to elucidate defence mechanisms in *Brassica* crops is systematically synthesised here. Major stresses confronting *Brassica* crop production and the metabolic basis of plant defence are first outlined. Current analytical platforms, including liquid chromatography–mass spectrometry, gas chromatography–mass spectrometry, ion mobility spectrometry, and mass spectrometry imaging, are critically evaluated alongside data processing workflows and multi-omics integration strategies. Key defence-related metabolite classes identified in *Brassica* crops, notably glucosinolates (GSLs) and their hydrolysis products, phenolic compounds, and lipid-derived signalling molecules, are surveyed with emphasis on their respective functions in biotic and abiotic stress responses. Metabolomics has been instrumental in revealing distinct metabolic reprogramming patterns triggered by diverse stresses, including pathogen infection, insect herbivory, drought, salinity, temperature extremes, and heavy metal stress. Metabolomics-informed crop improvement strategies, including marker-assisted breeding, genetic and metabolic engineering, and precision agronomic practices, are discussed together with current technical bottlenecks and future directions involving artificial intelligence, metabolic modelling, and spatial metabolomics. The compiled knowledge provides a comprehensive reference for leveraging metabolomics to enhance stress resilience and sustainable production of *Brassica* crops.

## 1. Introduction

The *Brassicaceae* family occupies a central position in global agriculture and economy, representing a major plant taxon. This family comprises approximately 4000 species, many of which are extensively cultivated as crops of considerable economic importance. Firstly, they serve as indispensable vegetable sources in the human diet, rich in beneficial secondary metabolites. Secondly, they constitute a vital source of oilseed crops, with a global annual production reaching tens of millions of tonnes. The genus *Brassica* is the most significant within the *Brassicaceae*, containing about 40 species with a wide distribution. The cultivated *Brassica* crops are primarily composed of six species, including three diploid species, *Brassica rapa* (*B*. *rapa*), *Brassica nigra* (*B*. *nigra*), and *Brassica oleracea* (*B*. *oleracea*), and three allopolyploid hybrids formed through natural chromosome doubling following interspecific hybridisation among the diploids, namely *Brassica juncea* (*B*. *juncea*), *Brassica napus* (*B*. *napus*), and *Brassica carinata* (*B*. *carinata*). Through long-term domestication, *Brassica* crops not only exhibit remarkable morphological diversity but also possess notable characteristics such as a large number of species, abundant genetic resources, extensive planting areas, and short growth cycles. The agricultural significance of this family is underscored by its most prominent oilseed crop, oilseed rape (*B. napus*). As one of the world’s leading oilseed commodities, oilseed rape plays a pivotal role in global edible oil production, animal feed supplementation, and the rapidly expanding biofuel sector. Its extensive cultivation across diverse agro-climatic zones, combined with its substantial share in international vegetable oil trade, makes it a key driver of agricultural economies and a strategic crop for food security and renewable energy policies in many countries.

However, the high and stable yields of *Brassica* crops are severely constrained by diseases such as *Sclerotinia sclerotiorum* (*S*. *sclerotiorum*) and *Plasmodiophora brassicae* (*P. brassicae*), as well as by biotic and abiotic stresses including insect pests, drought and heat stress [1]. Abiotic stresses exert significant inhibitory effects on the growth and yield of *Brassica* crops, with the degree of impact varying according to stress type, intensity, and crop genotype [2,3]. Drought stress is one of the most important factors restricting the production of *B*. *napus*, causing at least 30% yield loss annually. When drought occurs during the reproductive growth stage, yield losses are particularly severe; water withdrawal at flowering can cause a yield reduction of 41.3%, while water withdrawal at the pod development stage leads to a reduction of 32.3% [4]. Multiple studies consistently indicate that drought stress can reduce seed yield of rapeseed (*B. napus*) by an average of 27% to 57%, with drought-tolerant varieties showing about a 27% reduction, while sensitive varieties may suffer up to a 57% reduction [5]. Another study reported that drought leads to an average seed yield loss of 40% [6]. In addition to yield reduction, drought also significantly inhibits plant growth, leading to decreased plant height and reduced dry matter accumulation [7], while inducing the accumulation of osmotic regulators such as proline and soluble sugars, as well as increased antioxidant enzyme activities to cope with oxidative stress [8]. Salt stress also poses a serious threat to the yield of *Brassica* crops. Studies have shown that treatment with 200 mM NaCl can reduce seed yield of *B. napus* by approximately 25–35% [9]. Salt stress mainly reduces biomass accumulation and seed yield by inhibiting nitrogen uptake and accumulation, and by altering carbon and nitrogen metabolic characteristics [10]. Heavy metal stress also causes significant yield losses. In high-cadmium soils, seed yield can decrease by 36–72% [11]. Aluminium (Al) toxicity, as a major factor limiting crop growth in acidic soils, firstly inhibits root elongation, which in turn affects water and nutrient uptake and ultimately leads to yield reduction, the degree of root growth inhibition under Al stress varies among varieties, and Al-tolerant varieties can alleviate toxicity by secreting organic acids, such as citric acid and malic acid to chelate Al^3+^ ions [12,13].

In recent years, with advances in analytical technologies, metabolomics has made significant progress in the study of defence mechanisms in *Brassica* crops, gradually shifting from descriptive analyses to in-depth mechanistic investigations, and playing a particularly important role in elucidating disease resistance and stress tolerance mechanisms. In response to pathogen infection, combined untargeted and targeted metabolomic analyses using an ultra-high-performance liquid chromatography system (UPLC) paired with a quadrupole time-of-flight mass spectrometer (UPLC-Q-TOF-MS/MS) and proton nuclear magnetic resonance (^1^H-NMR) have demonstrated that resistant lines of *B. oleracea* restrict nutrient supply to *Xanthomonas campestris pv. campestris* during early infection by suppressing carbohydrate metabolism, while preferentially inducing the accumulation of indolic glucosinolates (GSLs), salicylic acid (SA), phenylpropanoids and phytoalexin precursors [14]. Under salt stress, integrated transcriptomic, proteomic and metabolomic analyses in *B. napus* have identified jasmonic acid (JA) and abscisic acid (ABA) as two key hormones regulating the salt stress response, with upregulation of BnPP2C phosphatases in the ABA pathway acting synergistically with upregulation of BnJAZ repressor proteins in the JA pathway to avoid excessive energy expenditure [15]. With respect to temperature stress, comparative metabolomics of *B. napus* varieties infected with Leptosphaeria maculans at 21 °C and 28 °C revealed that lipid metabolism, amino acid metabolism, carbohydrate metabolism and aminoacyl-tRNA biosynthesis were upregulated in resistant plants at 21 °C but downregulated at 28 °C, suggesting that high temperature compromises resistance by interfering with signal transduction networks [16]. However, current research on defence metabolomics in *Brassica* crops still faces a prominent issue of knowledge fragmentation [17]. Relevant studies are scattered across different species (*B. rapa*, *B. napus*, *B. juncea* and *B. oleracea*, among others), different stress types (biotic and abiotic), different tissue types, different analytical platforms and different metabolite identification levels. This fragmentation has resulted in an incomplete overall understanding of the defence metabolic regulatory networks in *Brassica* crops. Therefore, there is an urgent need for systematic integration and critical review of the above multidimensional metabolomic research findings, in order to reveal the commonalities and specificities of defence metabolism among different *Brassica* crops, to elucidate the general principles and specific responses of metabolic reprogramming under various stress conditions, and to evaluate the impact of different analytical platforms and metabolite identification strategies on research conclusions [18,19]. This review aims to systematically summarise the progress in the application of metabolomics to defence responses of *Brassica* crops, focusing on the three major classes of key defensive metabolites, namely GSLs and their hydrolysis products, phenolic compounds, and lipid-related metabolites integrating metabolic reprogramming patterns under both biotic and abiotic stresses, and discussing metabolomics-based crop improvement strategies and future directions, with the goal of providing a comprehensive reference for enhancing stress tolerance in *Brassica* crops and achieving sustainable production through metabolomics.

## 2. Methodological Approaches in Metabolomics

### 2.1. Technical Characteristics and Applicability of Major Analytical Platforms

**Figure 1:**
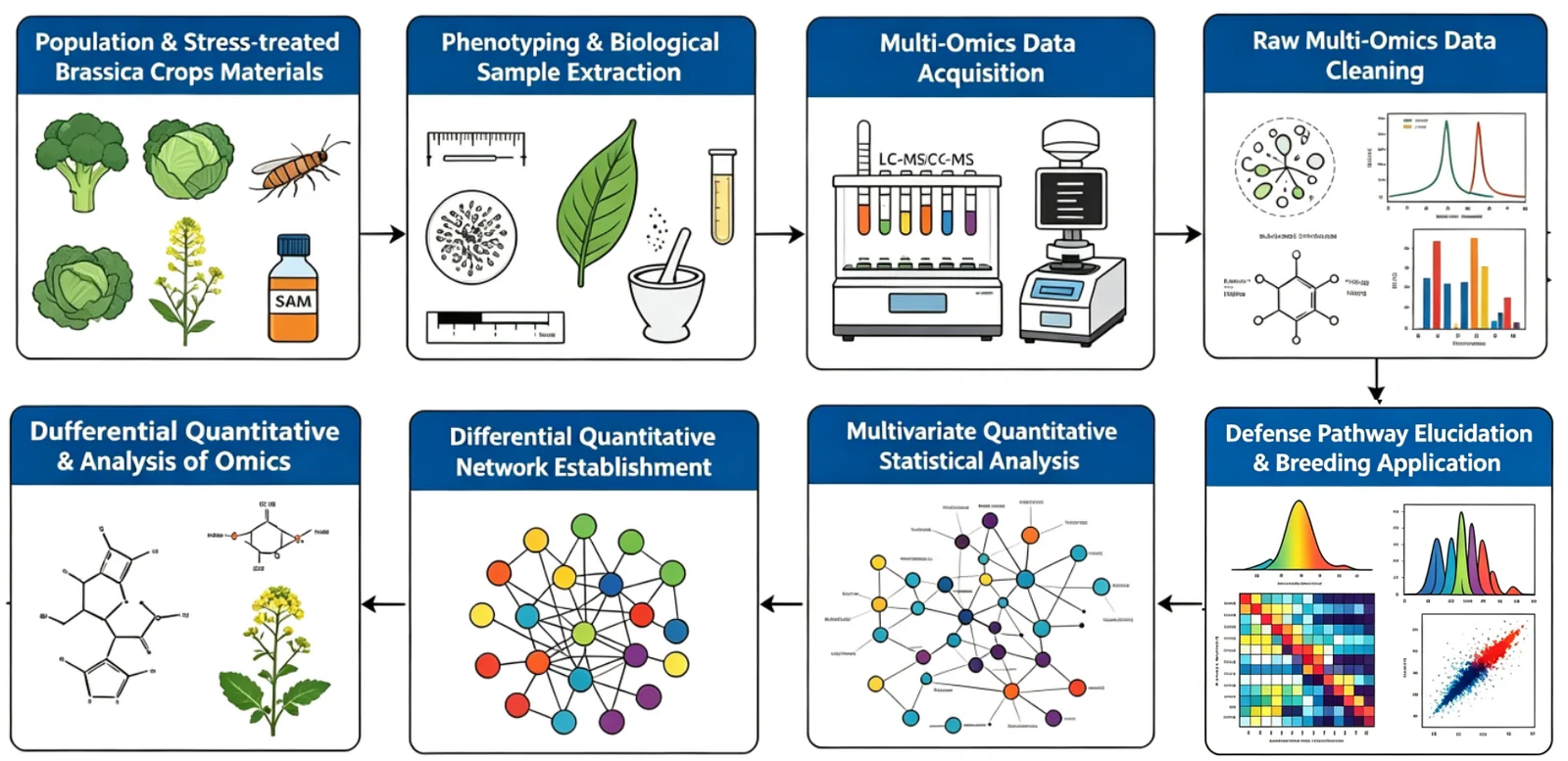
The workflow of research on plant defence response in *Brassica* crops. First, *Brassica* crop materials subjected to biotic or abiotic stress treatments are selected for systematic phenotypic characterisation and biological sample collection. Subsequently, metabolites are extracted from the collected samples, and multi-omics raw data are acquired using analytical platforms such as LC-MS and GC-MS. On this basis, strict quality control and data cleaning are performed on the raw data to ensure the reliability of subsequent analyses. Following data preprocessing, differential quantification analysis and multivariate statistical analysis are carried out to screen for differential metabolites and key regulatory molecules associated with defence responses. Furthermore, multi-omics data are integrated to construct gene–metabolite regulatory interaction networks, and defence regulatory pathways are systematically dissected through bioinformatics approaches. Finally, the analytical results obtained are applied to resistance breeding practices in *Brassica* crops, providing theoretical foundations and molecular targets for the development of new varieties with durable resistance.

Metabolomics aims to systematically resolve the overall composition and dynamic changes in all small-molecule metabolites in biological samples, relying on core technical methods using high-resolution, high-sensitivity analytical platforms. The workflow typically begins with rapid sample collection and metabolite extraction, followed by chromatographic separation and mass spectrometric detection to obtain raw spectra. Subsequent bioinformatics processing, including peak alignment, normalisation, and multivariate statistical analysis, leads to the selection and identification of differential metabolites. Finally, structural identification of candidate metabolites is performed using databases and standards, thereby revealing the metabolic response mechanisms of organisms under specific physiological or stress conditions (Figure 1).

Metabolomics research primarily relies on high-throughput analytical chemistry platforms such as liquid chromatography-mass spectrometry (LC-MS), gas chromatography-mass spectrometry (GC-MS), and nuclear magnetic resonance (NMR) spectroscopy. In recent years, emerging technologies including ion mobility spectrometry (IMS), mass spectrometry imaging (MSI), and capillary electrophoresis-mass spectrometry (CE-MS) have also progressively expanded the research boundaries of the field [3,20]. The main technical characteristics, advantages and limitations of these platforms are compared in Table 1. Among these, LC-MS is the most commonly used platform in plant metabolomics owing to its broad metabolite coverage, and is particularly suitable for the analysis of thermolabile, non-volatile or high-molecular-weight secondary metabolites such as phenolics, flavonoids, alkaloids and GSLs [21]. In studies of defence mechanisms in *Brassica* crops, LC-MS has been employed to dissect metabolic profiles under stress from *S*. *sclerotiorum* and *P*. *brassicae*, for example, by integrating transcriptomics and metabolomics to reveal resistance pathways in *B*. *napus* [22], or to identify key metabolites associated with clubroot resistance in *B*. *rapa* [23]. However, LC-MS still faces challenges in metabolite identification, including incomplete spectral characterisation and insufficient coverage of plant-specific databases. In contrast, GC-MS offers high resolution and good reproducibility for volatile and derivatised small-molecule metabolites; the fragment spectra generated by its electron ionisation source facilitate matching against standard libraries, and it is commonly used to detect signalling molecules such as terpenes and leaf volatiles. In oilseed rape, untargeted GC-MS metabolomics has been used to dissect the metabolic features of guard cells in response to ABA, successfully identifying various secondary metabolites such as GSLs and flavonoids [24]. Nevertheless, the requirement for sample derivatisation and its unsuitability for thermolabile substances limit its detection range. NMR spectroscopy serves as a complementary tool, providing detailed structural information through its non-destructive nature, low bias and excellent quantitation capability, and is particularly valuable for elucidating unknown compounds. Despite its relatively low sensitivity, it holds unique value in high-throughput metabolic phenotyping and structural confirmation [20]. In studies of black rot disease in *B*. *oleracea*, NMR combined with UPLC-Q-TOF-MS/MS revealed metabolic biomarkers [14]. With regard to emerging technologies, IMS introduces ion mobility separation prior to mass spectrometry, effectively distinguishing isomers, and its collision cross section values are becoming identification criteria [21], although IMS databases remain incomplete [25]. Ultra-performance liquid chromatography-travelling wave ion mobility-quadrupole time-of-flight mass spectrometry (UPLC-TWIMS-QTOF-MS) has been used for the identification of pesticide metabolites in *Brassica* crops, overcoming the limitations of spatial analysis, enabling in situ visualisation of metabolite distributions. For example, matrix-assisted laser desorption/ionisation mass spectrometric imaging (MALDI-MSI) has been applied to map flavonoid distribution in *B*. *napus* stems [26], and infrared matrix-assisted laser desorption ionisation (IR-MALDESI MSI) has been used for topographic imaging of *B*. *oleracea* leaves [27]. However, MSI still faces profound challenges in spatial resolution, sensitivity and quantitation [28]. CE-MS offers high sensitivity and resolution for analysing highly polar ionic metabolites such as phosphorylated sugars and organic acids, and is suitable for thermolabile compounds. Nevertheless, it suffers from a scarcity of reference spectral libraries, difficulty in achieving high-throughput analysis, and optimal performance only for small-volume samples [29]. Overall, the above platforms have each been applied to varying extents in studies of defence mechanisms in *Brassica* crops, but data standardisation across platforms, metabolite identification coverage and multi-omics integration remain major bottlenecks. Systematic comparative analyses of the advantages and limitations of each platform within this specific research context are still lacking, and future multi-platform synergistic applications are expected to provide a more comprehensive perspective for deciphering defence mechanisms in *Brassica* crops.

Although the aforementioned platforms have greatly expanded the technological capabilities of plant metabolomics, when the research objective shifts from metabolite screening to the accurate quantitation of specific defensive compounds, the choices of sample preparation, derivatisation, detection mode and other methodological parameters can have a substantial impact on the final biological conclusions. This is exemplified by the determination of two of the most representative and specific defensive metabolites in *Brassica*: GSLs and sinapoyl malate. GSLs and sinapoyl malate are two representative classes of unique defensive metabolites in *Brassica* crops. However, the methodological choices for the analysis of these two compound classes can significantly affect the resulting biological interpretations. For GSLs, quantitative analysis presents two distinctly different technical approaches: direct detection of intact GSLs versus indirect detection after desulphation [30,31]. Employing high-performance liquid chromatography with ultraviolet and diode array detector (HPLC-UV-DAD) combined with Sephadex-A25 column desulphation gives rise to varied desulphation efficiencies for individual GSLs, where incomplete desulphation can cause overestimation of rapidly desulphated forms and underestimation of slowly desulphated ones, whereas direct LC-MS/MS analysis of intact GSLs circumvents these issues, though it necessitates more sophisticated instrumentation [32,33]. More critically, once plant tissue is damaged, GSLs are rapidly hydrolysed by endogenous myrosinase, and the formation of hydrolysis products is highly dependent on factors such as pH [34]. If sample preparation fails to fully inactivate myrosinase prior to extraction, the detected entities will be hydrolysis products rather than the authentic intact glucosinolate profile, leading to serious misinterpretation of the plant’s defensive potential [32,35]. Similarly, the detection of sinapoyl malate also faces methodological challenges: UPLC-DAD-MS/MS methods can simultaneously quantify sinapic acid derivatives in a single chromatographic run, but if different extraction solvents are used or if internal standard correction is omitted during sample preparation, extraction efficiency and matrix effects may cause quantitative results to differ by several-fold between different studies [36,37]. In summary, seemingly minor methodological differences, such as extraction protocol, myrosinase inactivation, analytical target, and internal standard selection, essentially determine the very chemical entities being measured; different methods yield different molecular forms, whose biological meanings are not interchangeable [38,39]. Consequently, results from different studies are often not directly comparable, and when interpreting and integrating the metabolomics literature on *Brassica* crops, methodological considerations must be treated as the primary factor for evaluation.

### 2.2. Data Processing and Analysis

Raw data generated from metabolomics experiments must undergo a series of standardised processing steps before being transformed into biologically meaningful information. This workflow typically begins with raw data format conversion, followed by steps including peak detection, peak alignment, deconvolution, and normalisation to eliminate systematic errors caused by instrument drift and sample preparation variations, ultimately generating a quantitative data matrix containing signal intensity information for each metabolite [40]. Metabolite identification is a core step in data processing, primarily performed by matching measured exact mass, retention time, and tandem MS fragmentation spectra with public databases such as METLIN, HMDB, MassBank, or in-house standard libraries. For compounds unique to *Brassica* crops, such as GSLs, where obtaining standards is difficult, putative annotation often relies on high-resolution MS data and fragmentation patterns reported in the literature [41]. After constructing the data matrix, multivariate statistical methods such as principal component analysis (PCA) and partial least squares discriminant analysis (PLS-DA) are widely applied for dimensionality reduction and pattern recognition of metabolomics data. PCA, as an unsupervised method, can visually display the overall distribution among samples and inter-group separation trends, while PLS-DA, a supervised method, effectively screens for key metabolites driving group differences and identifies significantly abundant metabolites in combination with statistical tests [42]. To further understand the biological functions of differential metabolites, they are typically mapped to public metabolic pathway databases for enrichment analysis. This analysis reveals the biological processes most perturbed by stress by calculating the enrichment degree of differential metabolites in specific metabolic pathways. For example, significant enrichment of the phenylpropanoid biosynthesis pathway and glucosinolate metabolism pathway under clubroot infection provides key clues for dissecting plant defence mechanisms [43].

### 2.3. Integrated Multi-Omics Strategies

**Figure 2:**
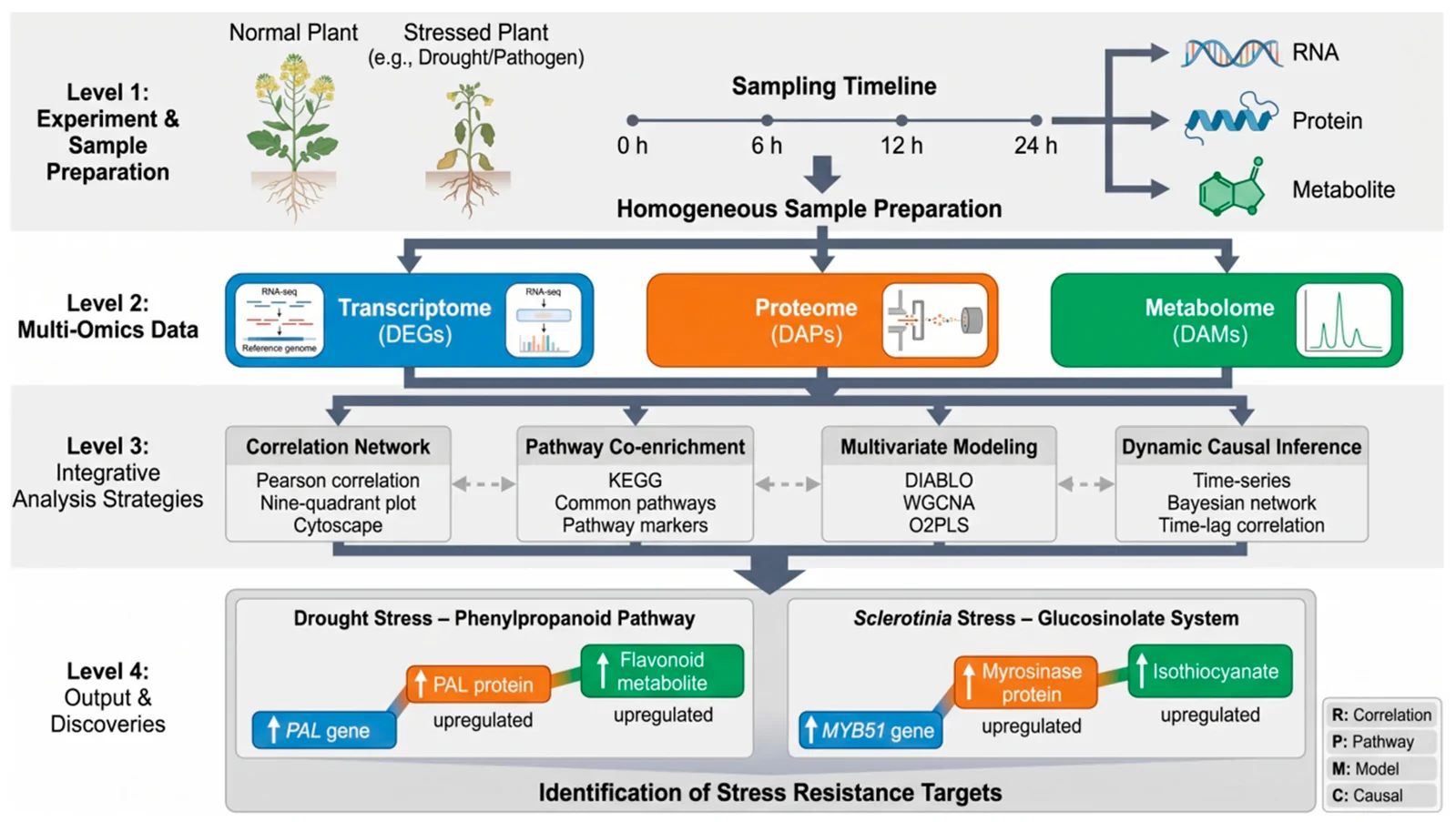
The metabolomics technology workflow and multi-omics integration strategy in *Brassica* crops. The workflow begins with controlled experiments comparing normal and stressed *Brassica* plants (e.g., under drought or pathogen attack) across a time-course sampling schedule (0, 6, 12, 24 h), followed by homogeneous preparation of samples for transcriptomic, proteomic and metabolomic profiling. The resulting multi-omics data (differentially expressed genes, differentially abundant proteins and differential metabolites) are then integrated through correlation network construction, pathway co-enrichment analysis, multivariate modelling and dynamic causal inference. This integrated framework enables the identification of stress-specific regulatory hubs, such as the upregulation of the *PAL* gene and protein in the phenylpropanoid pathway under drought, or the coordinated increase in *MYB51*, myrosinase, isothiocyanates and flavonoids in the glucosinolate system upon Sclerotinia infection. Ultimately, the pipeline yields validated pathway markers, causal relationships and predictive models that provide molecular targets and theoretical foundations for resistance breeding and metabolic engineering in *Brassica* crops. Abbreviations: DEGs, differentially expressed genes; DAPs: differentially accumulated proteins; DAMs: differentially accumulated metabolites; PAL: phenylalanine ammonia-lyase; KEGG: Kyoto Encyclopedia of Genes and Genomes; WGCNA: weighted gene co-expression network analysis; O2PLS: two-way orthogonal partial least squares; DIABLO: data integration analysis for biomarker discovery using latent variable approaches for omics studies.

Although single metabolomics analysis can provide phenotypic information at the metabolic level, it is difficult to fully reveal the complete regulatory network from genes to metabolites. Integrating metabolomics with other omics data has become a mainstream strategy for systematically dissecting complex biological processes in plants (Figure 2). Untargeted metabolomics, targeted metabolite profiling, conventional biochemical assays, phytohormone quantification, transcriptomic analysis and physiological validation constitute a multi-level analytical framework in studies of defence responses in *Brassica* crops, spanning from gene expression potential to physiological phenotype output. Transcriptomic analysis detects changes in transcript abundance under stress conditions, revealing the co-regulatory networks of transcription factors, signal transduction components and enzyme genes in metabolic pathways, and reflecting the plant’s preparedness to respond at the gene expression level [18,44]. Untargeted metabolomics aims to detect all detectable metabolites in a sample as comprehensively as possible, including numerous unknown compounds, and generates a full-spectrum metabolic fingerprint under stress by detecting hundreds to thousands of metabolites in a single run, it is particularly suitable for discovering unknown metabolites and screening differential metabolic biomarkers [3,20], although its limitation lies in the fact that a large number of spectral features cannot be effectively identified [45]. Targeted metabolite profiling and phytohormone quantification focus on pre-selected specific compound classes; the former enables precise quantitation of defence-related metabolites such as GSLs, phenolic acids and flavonoids using authentic standards [14], while the latter relies on high-sensitivity platforms such as UPLC-MS/MS to track the spatiotemporal dynamics of trace-level phytohormones, including JA, SA and ABA [46]. Together, they fulfil the function of precise quantitation of key molecules. Conventional biochemical assays focus on specific physiological and biochemical indicators such as malondialdehyde content and superoxide dismutase activity to verify the activity status of particular metabolic pathways or to assess the degree of stress-induced damage [47]. Physiological validation, through macroscopic parameters such as photosynthetic rate, relative water content and chlorophyll content, directly reflects the final health status and adaptation level of plants under stress conditions [48]. These approaches are not mutually substitutive but rather form a functionally complementary continuum from the micro to the macro scale: transcriptomics provides upstream regulatory drivers, untargeted metabolomics is responsible for global discovery, targeted analysis and phytohormone quantification achieve accurate quantitation of key molecules, conventional biochemical assays verify the activity of specific pathways, and physiological validation connects all molecular-level changes to the overall stress-tolerance phenotype of the plant. In systematic studies of defence responses in *Brassica* crops, the rational combination and data integration of these methods represent a critical pathway towards a comprehensive understanding of stress response mechanisms.

In a study on aluminium stress in *Brassica* crops, researchers successfully screened 138 core genes associated with aluminium tolerance by integrating quantitative trait locus (QTL) mapping, RNA sequencing (RNA-seq), and metabolomics analysis. These genes were mainly enriched in lipid metabolism, carbohydrate metabolism, and secondary metabolite biosynthesis pathways, and their expression patterns showed significant correlations with the accumulation levels of corresponding metabolites, thus constructing a complete regulatory chain from genotype to chemical phenotype [44]. Under salt stress, multi-omics analysis revealed synergistic regulation of pathways including plant hormone signal transduction, phenylpropanoid biosynthesis, and amino acid metabolism in rapeseed seedlings. The study found crosstalk between JA and ABA signalling pathways under salt stress, where upregulation of JAZ repressor proteins in the JA pathway synergistically interacts with activation of PP2C phosphatases in the ABA pathway to jointly regulate plant energy consumption and defence responses [15]. In clubroot resistance research, integration of transcriptomic and metabolomic data revealed synergistic upregulation of genes related to SA and JA/ethylene (ET) signalling pathways in resistant lines, accompanied by significant accumulation of defensive phenolic metabolites such as caffeic acid, protocatechuic aldehyde, and kaempferol [2]. These findings not only elucidate the interplay between hormonal signalling and secondary metabolism but also provide potential functional markers for resistance breeding.

## 3. Key Defence-Related Metabolites Identified in *Brassica* Crops

### 3.1. GSLs and Their Hydrolysis Products

GSLs and their hydrolysis products are characteristic sulphur-and nitrogen-containing secondary metabolites of *Brassica* crops, playing important roles in resistance to pests and diseases and in coping with environmental stresses [49,50] (Table 2). Based on their precursor amino acids, they are mainly classified into aliphatic, indolic, and aromatic GSLs, with representative compounds including sinigrin, glucoraphanin, and glucobrassicin, among others [51]. The biosynthesis of these compounds involves three key steps: side chain elongation, core structure formation, and secondary modifications, catalysed by various enzymes including methylthioalkylmalate synthase, cytochrome P450 enzymes, glutathione S-transferases, and sulfotransferases, and is centrally regulated by MYB family transcription factors [52]. Furthermore, the biosynthesis process is significantly influenced by genetic background, environmental factors, and agronomic practices, with sulphur deficiency reducing GSL accumulation, and abiotic stresses such as drought, high temperature, and salinity can induce increases in GSL content. Simultaneously, biotic stresses such as pathogen infection upregulate related genes such as *CYP79B2* and form complex regulatory networks through hormone signalling pathways like JA and SA, thus dynamically regulating the specific distribution of GSLs across different tissues and species [53].

In the plant defence system, intact GSLs themselves are biologically inactive, but upon tissue damage, activation of the endogenous enzyme myrosinase hydrolyses them into active products such as isothiocyanates, nitriles, and thiocyanates [41]. These hydrolysis products exert multiple functions in resisting insects and pathogens. On one hand, compounds such as isothiocyanates have repellent and toxic effects on herbivorous insects [52] and can directly inhibit bacterial and fungal growth, restricting pathogen invasion by inducing oxidative stress and membrane damage [93]. On the other hand, they can act as signalling molecules to activate systemic defence in plants, for example, by enhancing the antioxidant system, promoting stomatal closure to limit pathogen entry, or attracting natural enemies for indirect defence. It is noteworthy that although GSL hydrolysis products play important roles in both biotic and abiotic stresses, for instance, mitigating oxidative damage through antioxidant mechanisms [94], certain specialist pathogens may have evolved corresponding detoxification mechanisms, partially weakening their defensive efficacy.

With respect to glucosinolate metabolism, integrated UPLC-MS/MS and transcriptomic analyses have revealed that infection by *S. sclerotiorum* induces upregulation of glucosinolate biosynthetic pathways in resistant lines of *B*. *napus*, while genes involved in ethylene, SA and JA signalling pathways are also highly expressed [54]. Similarly, following infection by *Alternaria brassicicola* on leaves of *B*. *rapa*, liquid chromatography–electrospray ionisation–mass spectrometry (LC-ESI-MS) detection showed significant accumulation of indolic GSLs (including glucobrassicin and neoglucobrassicin), and their hydrolysis products exhibited over 70% inhibition of pathogen spore germination [53]. In broccoli (*B.oleracea* var. *italica*), combined transcriptomic and targeted metabolomic analyses further confirmed that the three indolic GSLs I3G, 4MI3G and 1MI3G all accumulated significantly in resistant lines, and the phenylpropanoid pathway was identified as one of the core metabolic pathways regulating resistance [60]. Beyond fungal diseases, bacterial and nematode stresses also induce remodelling of glucosinolate metabolism; infection by *Xanthomonas campestris pv. campestris* induces coordinated accumulation of indolic and aliphatic GSLs in leaves of *B*. *oleracea*, and their hydrolysis products, isothiocyanates and indole-3-carbinol, exhibited clear antibacterial activity in in vitro disc diffusion assays [49]. While infection by root-knot nematode (*Meloidogyne incognita*) in roots of *B*. *rapa* led, as shown by UPLC-MS/MS analysis, to a 75% increase in indolic GSLs and a 10–25% decrease in aliphatic GSLs, accompanied by a 1.5-fold increase in JA-Ile accumulation, indicating a systemic remodelling of the root glucosinolate defence profile [65]. Furthermore, HPLC-MS detection has also revealed that insect herbivory can induce accumulation of indolic GSLs and inhibit larval relative growth rate, confirming the direct antifeedant function of GSLs in defence against insects [64]. With regard to abiotic stresses, prolonged high-temperature stress suppresses the synthesis of indolic GSLs while causing passive accumulation of aliphatic GSLs, thus disrupting the defensive balance between the two classes [58]. Whereas low-temperature and freezing stresses specifically activate the accumulation of aliphatic and indolic GSLs, respectively [59]. UV-B radiation regulates glucosinolate metabolism in an intensity-dependent manner; low-intensity radiation upregulates the accumulation of indolic GSLs, whereas high-intensity radiation selectively induces the production of aromatic GSLs [67]. Exogenous hormone treatment experiments further reveal that indolic GSLs represent a core metabolic responsive node of the JA and SA signalling network [63].

Glucosinolate hydrolysis products also serve important defensive functions. Myrosinase activity assays combined with metabolite analysis have demonstrated that isothiocyanates (ITCs) accumulate transiently following tissue damage and exhibit stomach toxicity and antifeedant activity against chewing and piercing-sucking insects [68,69]. GC-MS analysis has shown that nitriles accumulate when the ESP pathway is enhanced, displaying specific antifeedant activity against specialist pests such as Pieris rapae [72,73]. HPLC-MS detection has indicated that indole-3-carbinol (I3C) is transiently generated by myrosinase-mediated hydrolysis of indolic GSLs and exhibits inhibitory effects against a range of fungi and bacteria [74]. Sulforaphane is one of the most representative bioactive isothiocyanate compounds derived from *Brassica* crops, produced by the hydrolysis of GSLs catalysed by myrosinase [95]. Sulforaphane is a potent chemopreventive agent against cancer, exerting anticancer effects through the modulation of both epigenetic and non-epigenetic pathways [96]. Its precursor, glucoraphanin, is widely distributed in *Brassica* crops such as broccoli, kale (*B.oleracea* var. *acephala*) and cabbage (*B.oleracea* var. *capitata*) [95]. Among these, broccoli sprouts represent the richest source of sulforaphane, containing 20 to 50 times more sulforaphane than mature broccoli [96]. The biosynthesis of glucoraphanin begins with the precursor amino acid L-methionine and comprises three key stages: side-chain elongation, core structure formation, and side-chain modification, involving the coordinated catalysis of multiple enzymes including methylthioalkylmalate synthase (MAM), cytochrome P450 enzymes (such as CYP79F1 and CYP83A1), glutathione S-transferase, sulfotransferase, and 2-oxoglutarate-dependent dioxygenase (AOP/GSL-ALK) [95]. At the transcriptional regulatory level, *MYB28* is the core transcription factor governing the accumulation of aliphatic GSLs, including glucoraphanin [97]. In Chinese cabbage (*B. rapa* subsp. *pekinensis*), three *BrMYB28* homologues (*BrMYB28.1*, *BrMYB28.2* and *BrMYB28.3*) all positively regulate glucosinolate biosynthesis, with overexpression of *BrMYB28.1* increasing total glucosinolate content by approximately five-fold [98]. Additionally, AOP (GSL-ALK) enzymes catalyse the conversion of glucoraphanin to other GSLs [5]. In terms of stress regulation, salt stress significantly increases sulforaphane content in broccoli sprouts; treatment with 160 mM NaCl elevated SF content by six-fold [70]. The BolMYB122-BolTGG1 module enhances salt tolerance in broccoli by accelerating glucosinolate hydrolysis to produce sulforaphane. Heat stress and hypoxia stress synergistically promote sulforaphane accumulation. Heat, hypoxia, and combined heat+hypoxia treatments increased sulforaphane content in 7-day-old broccoli sprouts by 153.73%, 95.15%, and 86.95%, respectively, compared with the control [95].

### 3.2. Phenolic Compounds

Phenolic compounds constitute another important class of secondary metabolites in *Brassica* crops, mainly comprising flavonoids, phenolic acids, tannins and lignin-related phenylpropanoid metabolites (Table 2). Their biosynthesis proceeds primarily through the phenylpropanoid pathway, with the expression of key enzymes such as phenylalanine ammonia-lyase, cinnamate-4-hydroxylase, chalcone synthase and chalcone isomerase directly influencing their accumulation [43]. The synthesis and accumulation of these compounds are finely regulated by genetic background and environmental stress. Under infection by *P. brassicae* (clubroot disease), combined metabolomic and transcriptomic analyses of roots of Chinese cabbage identified 257 metabolites associated with resistance, which were mainly enriched in the phenylpropanoid biosynthetic pathway [79]. Concurrently, in clubroot-resistant turnip (*B. rapa*) varieties, genes involved in JA, ET and brassinosteroid signalling pathways were coordinately upregulated, accompanied by significant accumulation of defensive phenolic metabolites such as caffeic acid, protocatechualdehyde and kaempferol [99].

With regard to flavonoids, UV-B radiation induces accumulation of flavonols such as kaempferol and quercetin in broccoli seedlings [35]. Flavonol biosynthesis is finely regulated by transcription factors such as *MYB* and *WRKY* [75]. Ultra-performance liquid chromatography-tandem mass spectrometry analysis revealed that feeding by *Plutella xylostella* led to differential accumulation of flavonoids in resistant cultivars of cabbage [77]. Concerning phenolic acids, salt stress activates the phenylpropanoid metabolic pathway in rapeseed seedlings, resulting in a significant increase in total phenolic acid content [78]. In terms of tannins, high-performance liquid chromatography-mass spectrometry detection indicated that condensed tannins accumulate most prominently in the seed coats of black-seeded rapeseed varieties [80,83], and these compounds play an important role in seed coat pigmentation [81,82].

In the plant defence system, phenolic compounds exert their functions through multiple mechanisms. Their core roles include antioxidant activity and direct antimicrobial effects, and by scavenging free radicals and chelating metal ions, phenolic compounds effectively alleviate oxidative damage [100], while at the same time they can directly interfere with pathogen enzyme systems, inhibiting their growth [101]. Overall, flavonoids, phenolic acids, tannins and lignin-related phenylpropanoid metabolites, together with defence compounds such as GSLs, constitute a complex chemical defence network in *Brassica* crops against both biotic and abiotic stresses.

### 3.3. Lipid-Related Metabolites

Lipid-related metabolites play an important role in the defence responses of *Brassica* crops (Table 2). Electrospray ionisation-tandem mass spectrometry lipidomic analysis has shown that during cold acclimation, the double bond index of membrane lipids in rapeseed increases slightly, a change that contributes to maintaining membrane fluidity and functional integrity at low temperatures [84]. Under salt stress, combined liquid chromatography-mass spectrometry and RNA-seq analysis revealed significant accumulation of unsaturated fatty acids in rapeseed roots, indicating that rapeseed adapts to salt stress by upregulating lipid metabolism to maintain cellular integrity and osmotic adjustment [85]. Furthermore, low-temperature stress significantly alters the fatty acid composition of seeds, and RNA-seq and metabolite analysis showed that under low night-time temperature, oleic acid content decreased, while erucic acid content increased. By modulating the degree of unsaturation and fluidity of membrane lipids, participating in stress signal transduction and regulating osmotic balance, lipid-related metabolites play an important role in the responses of *Brassica* crops to abiotic stresses such as low temperature and salinity.

### 3.4. Other Metabolites

Other defence-related metabolites mainly include alkaloids, terpenoids, sterols, and volatile organic compounds (VOCs), which play auxiliary roles in the response of *Brassica* crops to stress [2]. With regard to alkaloids, studies have suggested that they may play a role in the defence response of *B.oleracea* against *Xanthomonas campestris pv. campestris* infection, and specific metabolic pathways involving alkaloids have been identified as potential key candidate pathways in the infection response [102]. Furthermore, scopolamine, a tropane alkaloid, has been demonstrated, through integrated transcriptomic and metabolomic analyses, to significantly alleviate cold stress damage and improve survival rates in *B. napus* seedlings, and this study identified over 8000 differentially expressed genes and more than 3000 differentially accumulated metabolites, revealing the molecular mechanisms by which scopolamine regulates cold tolerance through carbon reallocation, cell wall reinforcement and reactive oxygen species scavenging [103]. With respect to sterols, multi-omics analysis of Chinese cabbage responding to black spot disease (*Alternaria brassicicola*) identified the sterol compound 4,4-dimethyl-5α-cholesta-7-en-3β-ol as a differential metabolite closely associated with defence responses [104]. The phytosterol composition in leaves of *B. napus* exhibits diversity due to genotype effects, and wild *Brassicaceae* germplasm also shows significant variation in phytosterol profiles; changes in individual phytosterols or their ratios may have important implications for plant tolerance [105].

Terpenoids encompass both structural and volatile types. Carotenoids, as terpenoid derivatives, participate in photoprotection and antioxidant functions, but their content often declines under stress conditions such as drought or waterlogging, while volatile terpenoids, including monoterpenes and sesquiterpenes, are important components of plant volatiles. The role of volatile terpenes as signalling molecules in plant defence is well documented. For example, oviposition by *Pieris brassicae* on *B. napus* triggers the release of specific volatiles such as α-pinene, dimethyl trisulfide and limonene, which can serve as early warning signals of herbivory for neighbouring undamaged plants [106]. Furthermore, although systematic studies on sterols in *Brassica* crops remain relatively limited, as structural components of cell membranes, sterols can indirectly support plant defence by maintaining membrane stability and participating in pathogen-associated molecular pattern-triggered immune responses.

VOCs are important chemical signalling molecules in plant defence responses [88], including green leaf volatiles, terpenes and sulphur-containing volatiles (Table 2). VOCs released by plants following herbivore feeding can serve as indirect defence signals, attracting parasitoid natural enemies to control pest populations [107]. For example, infection by Turnip mosaic virus significantly alters the composition of VOCs in leaves of *B. rapa*, with gas chromatography-mass spectrometry analysis identifying multiple differentially accumulated terpenes, green leaf volatiles and aromatic compounds [89]. However, the ecological function of volatile organic compound signals is susceptible to interference from environmental factors; atmospheric oxidative pollutants such as ozone can degrade VOCs, thereby disrupting their function as chemical signals and interfering with accurate host location by herbivorous insects [108].

In summary, alkaloids, terpenoids, sterols and VOCs participate in the defence responses of *Brassica* crops through direct or indirect mechanisms, acting synergistically with major defence systems such as GSLs and phenolic compounds to constitute a complex chemical defence network. Nevertheless, the specific biosynthetic regulatory pathways of these metabolites and their quantitative contributions to the overall defence system of *Brassica* crops require further investigation.

## 4. Applications of Metabolomics in Stress Response Studies

### 4.1. Biotic Stress Responses

Biotic stresses primarily include the effects of biological factors such as insect herbivory and pathogen infection on plants. Metabolomics, through untargeted or targeted analyses, reveals the metabolic reprogramming processes, key metabolic markers, and regulatory pathways in plants responding to biotic stress. Insect herbivory triggers specific metabolic responses in *Brassica* crops. Comparative metabolomics studies on *Plutella xylostella* infestation have elucidated the early defence response mechanisms in *B*. *napus*, revealing that tolerant lines accumulated greater amounts of benzenoids, lipids, organic acids and phenolic compounds following infestation, with the phenylpropanoid biosynthesis and ABC transporter pathways being significantly activated [109]. Furthermore, combined targeted metabolomic and transcriptomic analyses have been employed to investigate the metabolic responses of kale following methyl jasmonate treatment and larval infestation by the cabbage looper [110]. Studies have demonstrated that glucosinolate content in *Brassica* crops is closely associated with insect resistance; glucosinolate degradation products are released upon tissue damage and exert direct toxicity or repellent effects on pests [111]. Glucosinolate contents in seeds and leaves differ among rapeseed varieties, and varieties with lower glucosinolate content tend to be more susceptible to infestation by pests such as aphids [112]. In *B.nigra*, dual infection by root-knot nematodes (*Meloidogyne* spp.) and root-lesion nematodes (*Pratylenchus* spp.) induces significant changes in the shoot and phloem metabolomes, and elevated levels of indolic GSLs and hydroxycinnamic acids are closely correlated with reduced aphid survival [113]. Insect feeding also alters the compositional profiles of plant VOCs; for example, herbivore-induced plant volatiles (HIPVs) released by cabbage following larval feeding by *Pieris brassicae* can prime defence responses in neighbouring undamaged plants, enhancing their attractiveness to the parasitoid wasp *Cotesia glomerata* upon subsequent attack [114]. Similarly, pest feeding activates the JA signalling pathway, promoting the synthesis of defensive metabolites including GSLs, phenolic compounds and proteinase inhibitors, which enhance plant resistance to insects through direct toxicity or by attracting natural enemies [115].

Pathogen infection also elicits significant alterations in the metabolic profiles of *Brassica* crops. Combined untargeted and targeted metabolomics (UPLC-Q-TOF-MS/MS and ^1^H-NMR) have revealed metabolic reprogramming in cabbage during black rot disease; resistant lines restrict bacterial nutrient supply by suppressing carbohydrate metabolism and preferentially induce the accumulation of defensive compounds including indolic GSLs, SA, phenylpropanoids and phytoalexin precursors [14]. Another integrated transcriptomic and metabolomic analysis of black rot disease in *B*. *napus* showed that the tryptophan metabolic pathway was enriched in both susceptible and resistant varieties, with indolic GSLs and indole-3-acetic acid (IAA) both accumulating significantly after infection, and exogenous application of IAA promoting disease development [116]. In clubroot disease research, combined metabolomic and transcriptomic analyses revealed that trigonelline accumulates significantly in susceptible *B*. *rapa*, exogenous trigonelline application exacerbates clubroot symptoms, and overexpression of its biosynthetic gene *BrNANMT* increases susceptibility, indicating that trigonelline can serve as a biomarker metabolite for clubroot infection [23]. Moreover, combined metabolomic and transcriptomic analyses of clubroot disease in Chinese cabbage identified 257 metabolites associated with resistance, enriched in the phenylpropanoid biosynthetic pathway [79]. In blackleg disease research, the effect of temperature on resistance in *B*. *napus* has been elucidated through comparative metabolomics: at 21 °C, pathways including lipid metabolism and amino acid metabolism were upregulated in resistant lines, whereas these pathways were downregulated under heat stress at 28 °C, suggesting that high temperature may compromise resistance by disrupting signal transduction networks [16]. Furthermore, multi-omics approaches integrating genomics, proteomics, transcriptomics and metabolomics have been employed to gain a comprehensive understanding of host–pathogen interactions between *B*. *napus* and multiple diseases including *Leptosphaeria maculans*, *P. brassicae*, *S. sclerotiorum*, *Hyaloperonospora parasitica*, *Alternaria brassicae* and *Albugo candida*. The specific metabolic biomarkers identified through these metabolomic studies not only hold promise for early disease diagnosis but also serve as targets for the development of novel biological control strategies.

### 4.2. Abiotic Stress Responses

Abiotic stresses include environmental factors such as drought, salinity, and heavy metals, to which plants respond through metabolic pathways including osmotic adjustment, antioxidation, and detoxification. Abiotic stresses, including drought, salinity, and heavy metals, represent environmental factors to which plants respond through metabolic pathways such as osmotic adjustment, antioxidant defence, and detoxification. Metabolomics enables the quantification of dynamic changes in relevant metabolites, thereby revealing plant adaptive mechanisms. With respect to drought stress, metabolomic analyses have demonstrated that drought significantly alters the metabolic profile of Indian mustard (*B*. *juncea*), with marked increases in osmoprotectants such as trehalose, glucose, sucrose, proline and valine [90]. In oilseed rape, drought stress induces early responses including proline accumulation and phytohormone metabolism of ABA and JA [117]. Comparative metabolomics of Chinese cabbage under mild and severe drought stress has further revealed significant differences between contrasting genotypes in sugar metabolism, amino acid metabolism and organic acid metabolism [118]. Regarding heavy metal stress, metabolomic studies on Chinese cabbage have found that reduced glutathione plays diametrically opposite functions under different cadmium concentrations; it reduces cadmium accumulation under high cadmium stress, whereas it promotes cadmium accumulation under low cadmium stress [119]. Under aluminium stress, roots of *B*. *napus* exude organic acid anions such as citrate and malate to chelate Al^3+^ ions. Integrated analyses combining QTL mapping, transcriptomics and metabolomics have identified 138 key genes responsive to aluminium toxicity in the root system of rapeseed seedlings, primarily involved in lipid, carbohydrate and secondary metabolite metabolism [44]. Furthermore, combined metabolomic and transcriptomic analyses of salt stress during the germination stage of *B*. *napus* have identified 175 differential metabolites, mainly amino acids, carbohydrates and organic acids [78]. These metabolites not only serve as biomarkers for screening stress-tolerant crop varieties, but also provide important clues for a deeper understanding of the adaptive mechanisms of *Brassica* crops to abiotic stresses.

### 4.3. Cross-Stress Interactions

When plants are subjected to multiple stresses simultaneously or sequentially, their metabolic networks undergo complex reconfiguration, and metabolomic analyses integrated with multi-omics data can reveal synergistic or antagonistic effects among different stress factors. In oilseed rape, the combined effect of drought stress and clubroot disease on seed metabolic profiles differs from that of individual stresses [120]. The metabolic profiling of 330 seed samples harvested from two rapeseed varieties grown under drought, clubroot pathogen infection, and their combination revealed that the combined stress induced a distinct regulatory pattern in seed metabolic responses. Clubroot disease itself, by inducing gall formation on infected roots, restricts water and nutrient transport within the plant, and when occurring together with drought, may exacerbate the water stress effect. The regulatory mechanisms of this stress combination on seed quality remain to be fully elucidated. Under salt stress, integrated transcriptomic, proteomic and metabolomic analyses in *B*. *napus* have revealed that the two key hormones in the response to salt stress are ABA and JA [15]. The 24 h time point represents a critical juncture at which the plant adjusts multiple metabolic pathways to avoid excessive energy expenditure from over-response to salt stress; up-regulation of BnPP2C phosphatases in the ABA pathway and generalised up-regulation of BnJAZ repressor proteins in the JA pathway act in concert. This coordinated regulation between hormonal signalling illustrates the plant’s strategy of balancing defensive investment and energy consumption through fine-tuned metabolic reprogramming under multiple stresses. Similarly, in broccoli, seed priming with a glucosinolate-rich broccoli extract significantly enhances plant performance under salt stress [121]. The primed plants exhibited dynamic transcriptional reprogramming in root tissues under salinity, involving hormonal signalling, aquaporin regulation and activation of ion transporters, while secondary metabolism was also modulated. These studies indicate that when *Brassica* crops face multiple stresses, metabolic network reprogramming often manifests as a moderate suppression of primary metabolism coupled with a synergistic optimisation of defensive secondary metabolite accumulation. For instance, primary metabolites such as sugars and amino acids provide the material basis for osmotic adjustment, while secondary metabolites including GSLs and flavonoids assume direct defensive functions, thereby maintaining sufficient defensive capacity while safeguarding basic growth and development. This metabolic plasticity not only constitutes an important physiological basis for *Brassica* crops to cope with complex field environments, but also provides a critical entry point for deciphering the multi-stress adaptive mechanisms of crops using metabolomic approaches.

## 5. Metabolomics-Informed Crop Improvement Strategies

### 5.1. Marker-Assisted Breeding

Metabolomics significantly enhances the efficiency of crop breeding by identifying metabolite biomarkers associated with important agronomic traits. Core methods include metabolome genome-wide association studies (mGWAS) and metabolite quantitative trait locus (mQTL) analysis, which reveal associations between metabolites and genes, providing a molecular basis for selecting superior varieties [122]. In *B*. *napus*, a marker metabolite-based multi-omics analysis conducted on metabolomic data from 388 natural accessions identified for the first time 137 marker metabolites significantly associated with thousand-seed weight, of which 21 markers were simultaneously significantly correlated with both thousand-seed weight and seed oil content. By constructing a triple-network comprising 133 metabolites, 731 quantitative trait loci and 3321 genes, this study further confirmed that the transcription factor BnaA03.TGA6 exerts a negative regulatory effect on thousand-seed weight [123]. Similarly, a genome-wide association study focusing on the floral metabolic profiles of 119 *B*. *napus* accessions under drought stress identified 60 candidate genes, of which 18 are involved in stress-induced pathways, phenylpropanoid pathways and flavonoid modification [124]. These data provide specific linked single nucleotide polymorphism markers and candidate gene resources for marker-assisted selection programmes. In studies of clubroot resistance in *Brassica* crops, combined proteomic and metabolomic analyses have revealed the differential molecular responses between clubroot-resistant and clubroot-susceptible near-isogenic lines of *B*. *napus* during the early stages of infection by *P. brassicae* [125]. In the resistant line, 2515 and 1556 differentially abundant proteins were identified at 1, 4 and 7 days post-infection, involving primary metabolites such as amino acids, fatty acids and lipids, as well as secondary metabolites including GSLs. Concurrently, proteins related to cell wall reinforcement also underwent significant changes. Notably, 4-methoxyglucobrassicin was upregulated in both resistant and susceptible plants, and may serve as a potential marker for resistance screening. The advantages of metabolite markers lie in their ability to directly reflect the physiological status of the crop, their relative insensitivity to environmental variation, and their capacity to improve breeding precision. Integration with multi-omics data such as transcriptomics and genomics further optimises marker selection in precision breeding [123,124]. In the context of quality improvement of *Brassica* crops, metabolic profiling has been employed to investigate the relative abundances of 23 glucosinolate compounds across four tissues of five different morphotypes of *B*. *oleracea*. By combining these data with five corresponding high-quality genome assemblies and mRNA-Seq expression data, 183 glucosinolate-related genes were identified, providing important insights for breeding varieties with customised glucosinolate profiles [126]. With the standardisation of metabolite detection workflows and the continuous improvement of databases, marker-assisted breeding is progressing towards high-throughput automation, thereby supporting the development of climate-resilient crop varieties.

### 5.2. Genetic Engineering and Metabolic Engineering

Genetic engineering and metabolic engineering aim to enhance the accumulation of defensive metabolites in crops by editing key metabolic pathway genes, thereby improving stress tolerance. Clustered regularly interspaced short palindromic repeats (CRISPR)/Cas9-based gene editing is the mainstream tool for achieving this goal, enabling precise modification of multiple genes [127]. In recent years, substantial progress has been made in the application of this technology in *Brassica* crops. In *B*. *napus*, targeted mutagenesis of the transcription factor *MYB28* using CRISPR/Cas9 can partially downregulate the biosynthesis of aliphatic GSLs [128], while editing of the glucosinolate transporter (*GTR*) genes can alter the distribution pattern of GSLs within the plant [129]. In *B*. *juncea*, simultaneous editing of multiple homologues of the *GTR1* and *GTR2* families achieved a substantial reduction in seed glucosinolate content, while maintaining relatively high glucosinolate levels in vegetative tissues such as leaves to safeguard plant defence. These *GTR*-edited lines performed no worse than, or even better than, the wild type in defence against *S. sclerotiorum* and *Spodoptera litura* [130]. Multiplex editing targeting the *AOP2* gene family has been employed for directed regulation of metabolic flux. In *B*. *juncea*, editing of five *BjuAOP2* homologues resulted in glucoraphanin accumulation of 41.60, 75.10, 59.21 and 27.64 μmol/g dry weight in sprouts, microgreens, seeds and leaves, respectively [131]. Furthermore, CRISPR/Cas9-mediated DNA-free editing of *BolMYB28* has been utilised to develop broccoli varieties enriched in glucoraphanin [132]. Beyond gene knockout, overexpression strategies are also effective. Overexpression of the *Arabidopsis thaliana*-derived genes *CYP79B2* and *CYP83B1* in flowering Chinese cabbage not only significantly increased the accumulation of indolic GSLs, but also enhanced resistance to *S. sclerotiorum* and *Spodoptera exigua* [62]. Similarly, overexpression of the glucosinolate biosynthetic genes *BnMAM1*, *BnCYP83A1* and *BnUGT74B1* in *B*. *napus* enhanced resistance to *S. sclerotiorum* and *Botrytis cinerea* [62,133].

Synthetic biology, through the design of artificial metabolic pathways, further expands the application potential of metabolic engineering [134]. By introducing exogenous genes or optimising endogenous regulatory networks, directed biosynthesis of specific metabolites can be achieved. For example, studies on the efficient biosynthesis of glucoraphanin in *Brassica* crops via genetic engineering have systematically reviewed potential engineering targets across three stages: side-chain elongation, core structure formation and side-chain modification [135]. In addition, transgenic broccoli lines have been designed to optimise the hydrolysis efficiency of glucoraphanin during cooking, allowing effective release of bioactive hydrolysis products at cooking temperatures up to 20 °C higher than those tolerated by the wild type. The combination of synthetic biology with metabolomic analysis enables real-time monitoring of metabolic changes, ensuring the safety and efficacy of transgenic crops. In the future, with continuous improvements in metabolic pathway databases and computational models, multi-omics data-guided CRISPR editing technologies will enable more precise regulation of branched metabolic pathways, avoidance of feedback inhibition, and enhanced engineering efficiency.

### 5.3. Agronomic Practice Optimisation

Metabolomics provides data support for precision fertilisation and biostimulant development by analysing metabolic changes in crops in response to environmental stresses, thereby improving resource use efficiency and crop resilience [136]. In *B*. *napus*, combined transcriptomic and metabolomic analyses have revealed the specific response profiles of roots under individual deficiency of each of six macronutrients (nitrogen, magnesium, phosphorus, sulphur, potassium and calcium) prior to the appearance of any visible phenotypic reactions, all macronutrient deficiencies had already elicited substantial modulations of both transcriptomes and metabolomes, and 38 biochemical compounds capable of distinguishing between different types of macronutrient deficiency were identified [137]. Nitrogen deficiency also induced remobilisation of amino acids from old leaves to young leaves in genotypes with high nitrogen-use efficiency [138]. These specific metabolites can serve as molecular indicators of nutrient deficiency, providing a basis for precision fertilisation. Under aluminium toxicity conditions, using a population of 138 recombinant inbred lines, combined QTL mapping, transcriptome sequencing and metabolomic assays identified 457 differentially accumulated metabolites and 138 core genes highly correlated with 30 important metabolites in the root system of *B*. *napus* seedlings, primarily involved in the metabolism of lipids, carbohydrates and secondary metabolites [44]. On this basis, targeted fertilisation strategies can be devised to alleviate aluminium toxicity, while markers such as malondialdehyde and superoxide dismutase activities can be used to monitor stress levels.

The development of biostimulants represents another important application area. Metabolomic techniques facilitate the screening and validation of the efficacy of microbial or compound-based biostimulants. For example, the combined application of the microbial biostimulant ProbioHumus and calcium significantly alleviated drought stress in *B*. *napus* [47]. In *Brassica* crops, exogenous SA treatment can enhance resistance to clubroot disease; foliar application of SA on susceptible *B*. *napus* reduced disease severity index and gall size [139]. This precision regulation approach based on metabolic feedback enables dynamic adjustment mechanisms and significantly enhances the sustainability of agricultural production.

## 6. Challenges and Future Perspectives

The application of metabolomics in *Brassica* crop defence research faces significant technical bottlenecks. The primary challenge is the standardisation of metabolite identification and the improvement of databases, especially for *Brassica*-specific metabolites [140]. Current analyses rely heavily on mass spectrometry, but due to the structural diversity and wide dynamic concentration range of these specific metabolites, along with incomplete coverage in public databases and lack of uniform annotation standards, identification accuracy is limited, and data comparability across studies is poor. Another pressing need is the development of spatial metabolomics and single-cell metabolomics technologies. Existing methods are mostly based on tissue homogenisation, failing to resolve the spatial distribution of metabolites at the cellular or subcellular level, such as the differential accumulation of defence metabolites in leaf epidermis versus mesophyll. Although techniques such as MSI have been applied, they are limited by resolution, sensitivity, and challenges related to the tissue structural features of *Brassica* crops and low metabolite extraction efficiency, making it difficult to accurately reveal metabolic heterogeneity under stress.

At the biological level, the core challenge lies in the dynamic dissection and functional validation of metabolic networks. Defence responses in *Brassica* crops involve complex interactions between hormone signalling and secondary metabolic pathways, and the metabolic network is highly dynamic with spatiotemporal specificity [99]. However, current research often provides static snapshots, with insufficient tracking of real-time changes in metabolic flux, and causal validation of metabolite functions heavily relies on transgenic or gene editing technologies, which are time-consuming and highly species-dependent in *Brassica* crops [141,142,143], limiting large-scale functional validation. On the other hand, the utilisation of intraspecific and interspecific metabolic diversity remains inadequate [144]. Significant differences in metabolite profiles exist among different *Brassica* species and varieties. This diversity is a valuable resource for stress breeding, but the genetic basis and mechanistic links to stress tolerance have not been systematically dissected, hindering the effective use of favourable alleles in precision breeding [52].

Future research should focus on the specific application of artificial intelligence and metabolic modelling in studying defence mechanisms of *Brassica* crops, with the aim of addressing current bottlenecks such as insufficient database coverage and limited flux analysis capabilities [145]. In AI-driven metabolite identification, deep learning algorithms are expected to significantly enhance the structural elucidation efficiency of unknown metabolites [140]. To tackle the challenge that a large number of LC-MS/MS spectral features in *Brassica* crops metabolomics studies cannot be effectively identified, deep learning models such as Spec2Class have already demonstrated the ability to predict the chemical class of plant secondary metabolites from LC-MS/MS spectra with 93% accuracy [146]. The construction of a dedicated deep-learning-based metabolite identification pipeline specifically for *Brassica* crops such as *B*. *napus* is expected to systematically expand the chemical space coverage of defence-related metabolites in this genus.

With regard to metabolic modelling and flux prediction, genome-scale metabolic network models combined with flux balance analysis provide a theoretical framework for simulating the dynamic changes in metabolic flux under stress conditions. The constraint-based metabolic model bna572^+^ of developing *B*. *napus* seeds has successfully integrated ^13^C-metabolic flux analysis, validating the expression of 78% of genes and 97% of reactions in the model [147]. An in silico compartmentalised metabolic model of *B*. *napus* central metabolism has also provided a computational platform for the systemic study of regulatory aspects of *Brassica* crop metabolism [148]. However, conventional flux balance analysis relies on steady-state assumptions and struggles to capture transient metabolic dynamics under stress. The proposal of a machine learning-flux balance analysis integrated framework offers a new approach to overcoming this limitation by introducing transient kinetics to achieve more accurate flux estimation in plant tissues [149]. In *Brassica* crops, the combination of deep learning and flux analysis holds promise for simulating the dynamic changes in biosynthetic flux of defensive metabolites such as GSLs and flavonoids under heat stress, drought and pathogen infection, thereby predicting key regulatory targets and providing theoretical guidance for metabolic engineering.

In the future, by constructing an integrated predictive framework for defence metabolism in *Brassica* crops, employing AI-driven metabolite identification to fill database gaps, machine learning-enhanced flux analysis to overcome steady-state limitations, and multi-omics association analysis to guide the selection of gene editing targets. We will be able to systematically enhance the stress tolerance of rapeseed and other *Brassica* crops, develop new varieties that combine disease resistance, stress tolerance, and high yield, reduce dependence on chemical pesticides, and promote the sustainable development of green agriculture.

## Figures and Tables

**Table 1:** Comparison of technical characteristics of major metabolomics analytical platforms in defence responses of *Brassica* crops.

Platform	Main Target Metabolites	Typical Application Scenarios	Strengths	Limitations	References
LC-MS	Thermolabile, non-volatile or high-molecular-weight secondary metabolites: phenolics, flavonoids, alkaloids, GSLs, etc.	Metabolic profiling under pathogen stress; identification of key metabolites associated with disease resistance.	Broad metabolite coverage and greatest versatility.	The plant-specific database is insufficiently covered.	[3,20,21,22,23]
GC-MS	Volatile organic compounds (VOCs), small-molecule metabolites amenable to derivatisation (terpenes, leaf volatiles, sugars, amino acids, organic acids, etc.).	Detection of signalling molecules; profiling of secondary metabolites including GSLs and flavonoids.	High resolution and good reproducibility.	Requires derivatisation; not suitable for thermolabile substances.	[3,20,24]
NMR	Structural information of all hydrogen-containing metabolites; used in combination with UPLC-Q-TOF-MS/MS for biomarker identification.	Structural elucidation of unknown compounds; high-throughput metabolic phenotyping; structural confirmation.	Provides detailed structural information.	Relatively low sensitivity.	[14,20]
IMS-MS	Isomers difficult to distinguish by conventional mass spectrometry (e.g., flavonoid and glucosinolate isomers).	Separation and identification of isomers.	Effectively discriminating isomers.	Databases still underdeveloped.	[3,21,25]
MSI	Spatial distribution of metabolites at tissue or cellular level (e.g., flavonoids).	In situ visualisation of metabolite distribution.	Enabling in situ visualisation of metabolite localisation.	Spatial resolution, sensitivity and quantitation remain challenging.	[3,26,27,28]
CE-MS	Highly polar ionic metabolites: phosphorylated sugars, organic acids, etc.	Detection of highly polar ionic metabolites.	High sensitivity and resolution; suitable for thermolabile compounds.	Sparse reference spectral libraries; difficulty in achieving high-throughput analysis.	[3,29]

**Table 2:** Defensive metabolites and stress responses identified in *Brassica* crops.

Metabolite Type	Metabolites	Species	Tissue	Stress	Platform	Change	Reference
Aliphatic GSLs	Glucoraphanin, Gluconapin, Progoitrin	*B*. *napus*	Leaf	*S.sclerotiorum*	UPLC-MS/MS	Up	[54]
	Glucoraphanin, Glucoiberin	*B*. *oleracea*.	Leaf	Boron deficiency	HPLC-MS	Up	[55]
	Total aliphatic GSLs	*B*. *oleracea*	Leaf	Sulphur deficiency	HPLC-MS	Down	[56]
	Total aliphatic GSLs	*B*. *rapa*.	Leaf	Phosphorus deficiency	HPLC	Up	[57]
	Total aliphatic GSLs	*B*. *napus*	Leaf	High temperature	LC-MS/HPLC-ESI-MS	Up	[58]
	Total aliphatic GSLs	*B*. *oleracea*.	Leaf	Low temperature	HPLC-DAD	Up	[59]
Indolic GSLs	Glucobrassicin, Neoglucobrassicin, 4-Hydroxyglucobrassicin	*B*. *rapa*	Leaf	*A*. *brassicicola*	LC-ESI-MS	Up	[53]
	Three indolic GSLs: I3G, 4MI3G, and 1MI3G	*B*. *oleracea*	Leaf	*A*. *brassicicola*	LC-MS	Up	[60]
	Neoglucobrassicin	*B*. *oleracea*	Leaf	*A*. *brassicicola*	LC-MS	Up	[61]
	Glucobrassicin, Neoglucobrassicin, 4-Methoxyglucobrassicin	*B*. *napus*	Leaf	*B*. *cinerea*	HPLC	Up	[62]
	Total indolic GSLs	*B*. *rapa*	Leaf, root	MeJA/SA treatment	HPLC	/	[63]
	Total indolic GSLs	*B*. *oleracea*	Leaf	*Pieris rapae*	HPLC	Up	[64]
	Indolic GSLs, aliphatic GSLs	*B*. *rapa*	Root	*M*. *incognita*	LC-MS/UPLC-MS/MS	Indolic, up; aliphatic, down	[65]
	Glucobrassicin, Neoglucobrassicin	*B*. *oleracea*	Leaf	Freezing	HPLC-DAD	Up	[59]
	Total indolic GSLs	*B*. *oleracea*	Leaf	*X*. *campestris*	HPLC/HPLC-MS	Up	[66]
	Total indolic GSLs	*B*. *napus*	Leaf	Low-intensity UV-B	HPLC	Up	[67]
Aromatic GSLs	Gluconasturtiin	*B*. *napus*	Leaf	High-intensity UV-B	HPLC	Up	[67]
Glucosinolate hydrolysis products	Isothiocyanates: Allyl-ITC, Benzyl-ITC	*B*. *oleracea*	Leaf, root	Enzymatic hydrolysis after tissue damage	/	Up	[68,69]
	Sulforaphane	*B. oleracea*	Sprouts	Salt	HPLC-MS	Up	[70]
	Sulforaphane	*B*. *oleracea*	Florets	Melatonin	LC-MS/MS	Up	[71]
	Nitriles: 3-Butenenitrile, Phenylpropionitrile	*B*. *rapa*	Leaf	Insect	GC-MS	Up	[72,73]
	Indole-3-carbinol	*B*. *oleracea*	Tissue	Pathogen/mechanical wounding	HPLC-MS	/	[74]
Flavonoids	Kaempferol, Quercetin, Isorhamnetin	*B*. *oleracea*	Shoot	UV-B	HPLC-MS	Up	[35,75,76]
	Kaempferol, Quercetin	*B*. *oleracea*	Leaf	*Plutella xylostella*	UPLC-MS/MS	Up	[77]
Phenolic acids	Total phenolic acids	*B*. *napus*	Seedling	Salt	LC-MS	Up	[78]
	Total phenolic acids	*B rapa*	Root	*P. brassicae*	UPLC-MS/MS	Up	[79]
Tannins	Proanthocyanidins	*B*. *napus*	Seed coat	Seed coat pigmentation	HPLC-MS	/	[80,81,82,83]
Saturated fatty acids/glycerophospholipids	membrane lipid: PA, PI, PE, LPC	*B*. *napus*	Leaf	Cold	ESI-MS/MS	Up	[84]
Unsaturated fatty acids	Linoleic acid, α-Linolenic acid, Oleic acid.	*B*. *napus*	Root	Salt	LC-MS	Up	[85]
Fatty acids	Oleic acid, Erucic acid	*B*. *napus*	Seed	LNT vs HNT	GC-MS	Oleic acid, down; Erucic acid, up	[86]
Phytoalexins	Brassinin, Cyclobrassinin, Spirobrassinin, Brassilexin	*B*. *rapa*	Leaf	CuCl_2_-induced	UPLC-HRMS/MS	Up	[17]
	Brassinin, Cyclobrassinin, Spirobrassinin,	*B*. *napus*	Root	*Pseudomonas aeruginosa*	LC-HRMS	Up	[87]
VOCs	Various terpenes, green leaf volatiles, aromatic compounds	*B*. *rapa*	Leaf	TuMV	GC-MS	/	[88,89]
Osmolytes	Proline, Trehalose, Glucose, Sucrose, Valine	*B*. *juncea*	Leaf	Drought+PGPR	GC-MS	Up	[90,91]
Phytohormones	ABA, JA	*B*. *napus*	Leaf	Salt	LC-MS/MS	/	[15]
	IAA, ABA, JA	*B*. *oleracea*	Stalk incision	Post harvest	LC-MS/MS	/	[92]

Summary of stress-induced metabolic changes in *Brassica* crops as determined by metabolomics platforms. Abbreviations: ITC, isothiocyanate; PA, phosphatidic acid; PI, phosphatidylinositol; PE, phosphatidylethanolamine; LPC, lysophosphatidylcholine; IAA, indole-3-acetic acid; MeJA, methyl jasmonate; TuMV, turnip mosaic virus, LNT, low nighttime temperature; HNT, high nighttime temperature; turnip mosaic virus; PGPR, plant growth-promoting rhizobacteria.

## Data Availability

All the necessary data are included in this manuscript.
